# Determination of Drug Compliance of Hypertensive Individuals

**Published:** 2017-01

**Authors:** Ezgi BAĞRIAÇIK, Buket ÇIMEN, SAĞDIÇ Tağrık, Ümran DAL YILMAZ

**Affiliations:** Dept. of Nursing, Faculty of Health Sciences, Near East University, Nicosia, Northern Cyprus

## Dear Editor-in-Chief

The majority of hypertensive individuals require drug therapy to control blood pressure levels. Lifestyle changes and regular use of prescribed medication can significantly contribute to controlling hypertension. A correlation between non-drug compliance and insufficient blood pressure control was noted amongst hypertensive individuals. Therefore, in order to minimize the risk of hypertension and other systemic diseases, drugcompliance is essential (1–3).

The current study was conducted to determine drug compliance amongst hypertensive individuals. Overall, 170 hypertensive participants selected for the current study were all residents of the Turkish Republic of Northern Cyprus (TRNC) and were all registered with the health department subsidiary of Municipality of Dikmen located in the district of Girne of TRNC. Prior to interview in each participant in the privacy of their own home, consent was obtained from both the Municipality of Dikmen and from each of the participants. Informed consent was also obtained from Near East University Ethics committee (YDU-244). The relevant information regarding the study was also given to each participant prior to commencing with the study.

Amongst the selected participants, 58.8% are female, 95.3% are married, and 48.2% are first school-graduates. More than half (63.5%) of the surveyed participants have other chronic diseases in addition to hypertension, diabetes mellitus was found to be the most common (34.1%) of these conditions. Of the hypertensive individuals involved in the study; 80% undergo medical examinations regularly, 45.9% has Body Mass Index (BMI) between 25–29.9 kg/m^2^ and an excess of weight gain was noted amongst participants. More than half of the participants (61.2%) restricted salt from their diet, while 85.7% had a protein-rich diet. Almost all of the individuals involved in the current study use their medication regularly, 89.4% have prior knowledge about the medication and 55.3% of these participants reported that a physician obtained the source of the information. Approximately half (50.6%) of the participants were unaware of the name of the medication used while 85.9% were unaware of possible side effects associated with the medication, even though 10.6% reported a cough. In the case when participants forgot to take their medication at the recommended time, 82.4% immediately took their medication once realizing. Almost none of the attendants (91.8%) measured blood pressure levels prior to taking their medication (Table 1).

**Table 1:** Features of drug use of hypertensive individuals

**Features (n:170)**	**n**	**%**
**Type of treatment**		
Regular drug users	170	100.0
**Information regarding the prescribed medication**		
Received	152	89.4
Not received	181	0.6
**Information regarding the prescribed medication was delivered by an individual or Institution (n:152)**		
Doctor	84	55.3
Nurse	10	6.6
Pharmacist	10	6.6
Prospectus	48	31.5
**Awareness of the name of the medication in use**		
Yes	84	49.4
No	86	50.6
**Awareness of the possible side effects of the prescribed medication**		
Yes	24	14.1
No	146	85.9
**Side effects reported**		
None	146	85.8
Headache	2	1.2
Dizziness	2	1.2
Cough	18	10.6
Weakness	2	1.2
**Dosage of prescribed medication**		
1×1	88	51.8
2×1	78	45.9
3×1	4	2.3
**In the case of failing to take medication at the recommended time, the medication was taken;**		
Immediately after realizing	140	82.4
1 hour after realizing	84.7	
Failed to take the medication	22	12.9
**Measuring blood pressure before taking the prescribed medication**		
Yes	14	8.2
No	156	91.8

Although individuals with hypertension are aware of recommended factors, which lead to a healthy lifestyle and the correct use of prescribed medication, the majority of participants was not employed these recommendations into their lifestyle and in turn, was ineffective in controlling their blood pressure levels. Therefore, individuals diagnosed with hypertension receive the correct information regarding hypertension, associated chronic conditions, effective medical treatments, and rehabilitation. Perhaps the routine involvement of nurses can improve the management of hypertension amongst individuals (4, 5).
